# Genomic Epidemiology of *Streptococcus pneumoniae* Isolated in a Tertiary Hospital in Beijing, China, from 2018 to 2022

**DOI:** 10.3390/pathogens12020284

**Published:** 2023-02-09

**Authors:** Shuaihua Fan, Ning Duan, Wenjing Chen, Xiuying Zhao, Lijun Wang, Pengcheng Du, Jun Guo

**Affiliations:** 1 Department of Pulmonary and Critical Care Medicine, Beijing Tsinghua Changgung Hospital, School of Clinical Medicine, Tsinghua University, Beijing 102218, China; 2 Department of Clinical Laboratory, Beijing Tsinghua Changgung Hospital, School of Clinical Medicine, Tsinghua University, Beijing 102218, China; 3 Department of Otolaryngology Head and Neck Surgery, Beijing Tsinghua Changgung Hospital, School of Clinical Medicine, Tsinghua University, Beijing 102218, China; 4 Beijing Key Laboratory of Emerging Infectious Diseases, Institute of Infectious Diseases, Beijing Ditan Hospital, Capital Medical University, Beijing 100015, China

**Keywords:** *Streptococcus pneumoniae*, sequence type, serotype, antimicrobial resistance, healthcare-associated infection

## Abstract

*Streptococcus pneumoniae* is one of the most common bacterial pathogens of a wide range of community-acquired infections. It has been more and more recognized that this bacterium could also play a role as a cause of nosocomial infections. In this study, by retrospective analysis of the phenotypic resistance characteristics and genomic characteristics of 52 *S. pneumoniae* isolates in a hospital in Beijing, China, from 2018 to 2022, we explored the carriage of resistance genes and mutations in penicillin-binding proteins corresponding to the resistances, and identified the population diversity based on the prediction of serotypes and identification of sequence types (STs). The isolates displayed resistances to erythromycin (98%), tetracycline (96%), sulfonamide (72%) and penicillin G (42%). Among the 52 isolates, 41 displayed multiple-drug resistance. In total, 37 STs and 21 serotypes were identified, and the clonal complex 271 serogroup 19 was the most prevalent subtype. Only 24 isolates (46.2%) of 7 serotypes were covered by the 13-valent pneumococcal conjugate vaccination. The isolates showed high carriages of resistance genes, including *tet*(M) (100%) and *erm*(B) (98.1%); additionally, 32 isolates (61.5%) had mutations in penicillin-binding proteins. We also observed 11 healthcare-associated infections and 3 cases infected by different subtypes of isolates. We did not find nosocomial transmissions between the patients, and these cases might be associated with the asymptomatic colonization of *S. pneumoniae* in the human population. Our results called for further active surveillance of these subtypes, as well as the continuous optimization of the treatment protocols.

## 1. Introduction

*Streptococcus pneumoniae* is one of the most common bacterial pathogens of a wide range of community-acquired infections, including respiratory infections, such as pneumonia, bronchitis and rhinitis, and invasive infections, such as meningitis, bacteremia and septicemia [1]. It is considered to be a major cause of death of children under 5 years old worldwide [2]. Moreover, it has been more and more recognized that this bacterium could also play a role as a cause of nosocomial infections [3]. The prevalence of *S. pneumoniae* is partially due to its wide carriage by healthy people on the mucosal surface of the upper airways, e.g., 20–60% of school-aged children and 5–10% of adults without children are healthy carriers [4].

*S. pneumoniae* are of large diversity. There are more than one hundred known serotypes and more than seventeen thousand sequence types (STs) [5]. The most prevalent serotypes include 19F, 23F, 14, 6B, 1, 19A and 3, and the most prevalent STs include ST199, ST320, ST81, ST271 and ST876 [6,7,8]. Currently, up to 13 serotypes are included in pneumococcal conjugate vaccines (PCVs). However, researchers have observed serotype replacement after the use of PCVs. The increased use of PCVs led to decreases in the serotypes covered by PCVs, and increases in the serotypes not in PCVs [9,10,11]. In addition, the recombination occurring in the gene cluster required for the capsular synthesis between different subtypes led to serotype switching [12], and the new strains might have advantages under the immune selective pressure constructed by the wide use of PCVs [13].

In 2017, the WHO included penicillin-non-susceptible *S. pneumoniae* as one of the 12 most critical, priority pathogens to guide the research, discovery and development of new antibiotics. The resistance of *S. pneumoniae* against ß-lactams and macrolides is a major concern worldwide [14]. In China, the proportion of penicillin-resistant *S. pneumoniae* (PRSP) is 86.9% in non-meningitis isolates in children [15]. The amino acid mutations occurring in penicillin-binding proteins (PBPs) result in decreased affinity for penicillin to inhibit the final steps of peptidoglycan synthesis by binding to the PBPs [14]. The macrolide resistance is most associated with two mechanisms: one is *erm*(B), encoding an enzyme that methylates 23S rRNA to induce target-site alteration; the other is *mef*(A) and *mef*(E), encoding active efflux pumps [16]. The increasing resistance is causing *S. pneumoniae* to become more dangerous in both community and healthcare settings.

In this study, by retrospective analysis of the phenotypic resistance characteristics and genomic characteristics of *S. pneumoniae* isolates in a hospital in Beijing, China, we explored the carriage of resistance genes and mutations corresponding to the resistances and identified the population diversity based on the prediction of serotypes and identification of STs. The clonal complex 271 serogroup 19 was the most prevalent subtype in our investigation, with high carriage of resistance genes and mutations in PBPs. Our results called for further active surveillance of these PRSP subtypes, as well as the continuous optimization of the treatment protocols.

## 2. Materials and Methods

### 2.1. Strain Collection and Associated Clinical Characteristics

We performed a retrospective study from July 2018 to July 2022 in Beijing Tsinghua Changgung Hospital, a Class A tertiary hospital in Beijing, China. Our hospital possesses 1000 beds and mainly provides services to the population of Beijing, having 24.1–39.1 thousand admissions each year in the last 5 years. *S. pneumoniae* isolates were cultured on a Columbia blood agar plate. The monoclonal isolates selected were first identified by MALDI-TOF mass spectrometry and then confirmed by the Vitek compact 2 system. All of the *S. pneumoniae* isolates were stored at −80 °C. The clinical characteristics, including the age, sex, underlying diseases, infection type, use of invasive devices and outcomes, were obtained from electronic medical records. We defined community-acquired infections (CAIs) as those that occurred before or within 48 h of admission, and healthcare-associated infections (HAIs) as those that occurred after 48 h following admission. This study was approved by the Beijing Tsinghua Changgung Hospital Ethics Committee (18,116-0-01), and the Guidelines for Human Experimentation (PRC) were followed throughout. Informed consent was not obtained for this study since clinical data were de-identified.

### 2.2. Antimicrobial Susceptibility Testing

The antimicrobial susceptibility testing was performed according to the 2020 Clinical and Laboratory Standards Institute (CLSI) guidelines, using the following agents: penicillin G, amoxicillin, cefotaxime, ceftriaxone, ertapenem, meropenem, levofloxacin, moxifloxacin, ofloxacin, erythromycin, telithromycin, linezolid, vancomycin, tetracycline, chloramphenicol and sulfonamide. The results were interpreted following the Clinical and Laboratory Standards Institute (CLSI) guidelines [17]. The multiple-drug resistance (MDR) phenotype was defined as being resistant to three or more different classes of antimicrobial agents.

### 2.3. Whole-Genome Sequencing and De Novo Assembly

The genomic DNA of all isolates was extracted with the TIANamp Bacteria DNA Kit (Cat. no. DP302, Tiangen, China), and then sequenced using the NovaSeq 6000 platform (Illumina, San Diego, CA, USA) by constructing paired-end libraries with an average insert size of 500 bp to obtain 150 bp reads. We used the fastp software (https://github.com/OpenGene/fastp, accessed on 18 November 2022) to obtain the clean data from the raw data, and assembled the clean data with SPAdes v3.15.2 [18]. The draft genome sequences were subsequently annotated using Prokka [19]. 

The multi-locus sequence types of the isolates were analyzed by comparing them against the reference database (https://pubmlst.org/organisms/streptococcus-pneumoniae, accessed on 18 November 2022) by BLAST [20]. The serotypes of the isolates were predicted based on the clean read data using PneumoCaT (https://github.com/phe-bioinformatics/PneumoCaT, accessed on 18 November 2022). Antimicrobial resistance genes, resistance-associated mutations, virulence genes and plasmid replicon types were annotated by comparison with relevant databases, including ResFinder, Virulence Factor Database, ISfinder and plasmidFinder [21,22,23], using the BLAST software [20]. We selected thresholds of 90% identity and minimum length coverage of 80% to identify antimicrobial resistance and virulence genes.

### 2.4. Phylogenetic Analysis

As part of our phylogenetic analysis, the sequencing reads were mapped to the reference sequence of *S. pneumoniae* ATCC700669 (accession no.: NC_011900) using Bowtie 2 v2.2.8 software, and the single-nucleotide polymorphisms (SNPs) were analyzed with Samtools v1.9 and combined using the iSNV-calling pipeline (https://github.com/generality/iSNV-calling, accessed on 18 November 2022) we previously constructed [24,25]. High-quality SNPs supported by more than five reads of mapping quality >20 were retained. The recombination sites were subsequently detected by Gubbins v2.4.1 software [26]. The concatenated sequences of filtered polymorphic sites conserved in all the isolates were used to perform phylogenetic analysis using the maximum likelihood method by IQ-Tree v2.1.2 software [27]. The best-fitting substitution model (GTR + F + ASC + R3) was identified using ModelFinder [28]. The consensus tree was constructed from 1000 bootstrap trees using UFBoot2 [29]. 

## 3. Results

### 3.1. Sources of the Isolates and Their Antimicrobial Susceptibility Profiles

**Figure 1:**
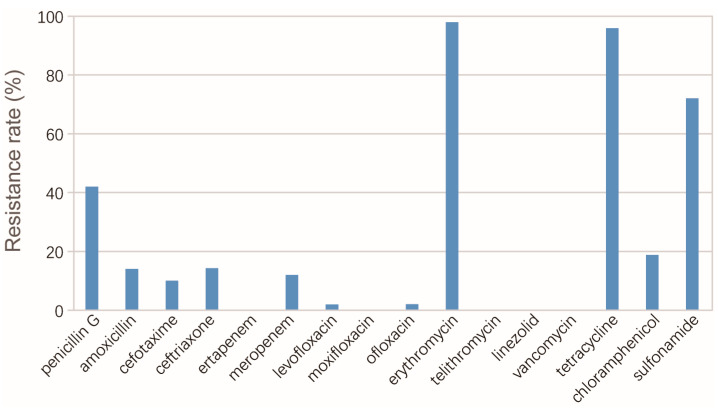
Resistance rates of *S. pneumoniae* isolates to the antimicrobials tested in this study.

We identified 52 *S. pneumoniae* isolates from 47 patients in total (Table 1). The samples from which the isolates were cultured were blood (8 isolates) and respiratory samples (44 isolates), including sputum (26 isolates), throat swab (10 isolates), bronchoalveolar lavage fluid (3 isolates) and bronchial aspirate (2 isolates). The information of 2 patients was missing, and the remainder included 37 males and 8 females, with a median age of 67 years (IQR: 6.5–74 year). Of these, 12 patients (26.7%) were 4–7-year-old children, and 27 patients (60%) were older than 60 years. The underlying diseases included sleep apnea, lung cancer, lung infection and cerebral infarction (Table 1). A total of 34 patients were diagnosed as CAIs, and 11 patients were diagnosed as HAIs. Among the seven cases with systematic infections of which the isolates were cultured from blood, three cases were CAIs and four were HAIs. Subjected to antimicrobial susceptibility testing, the isolates displayed resistance to multiple classes of antimicrobials, including erythromycin (98%), tetracycline (96%), sulfonamide (72%) and penicillin G (42%) (Figure 1). Among the isolates, 41 (78.8%) were identified as MDR and 21 (40.4%) were PRSP. We observed a decrease in resistance rate and minimum inhibitory concentration over the years, particularly in 2022.

### 3.2. Molecular Characteristics of the Isolates

**Figure 2:**
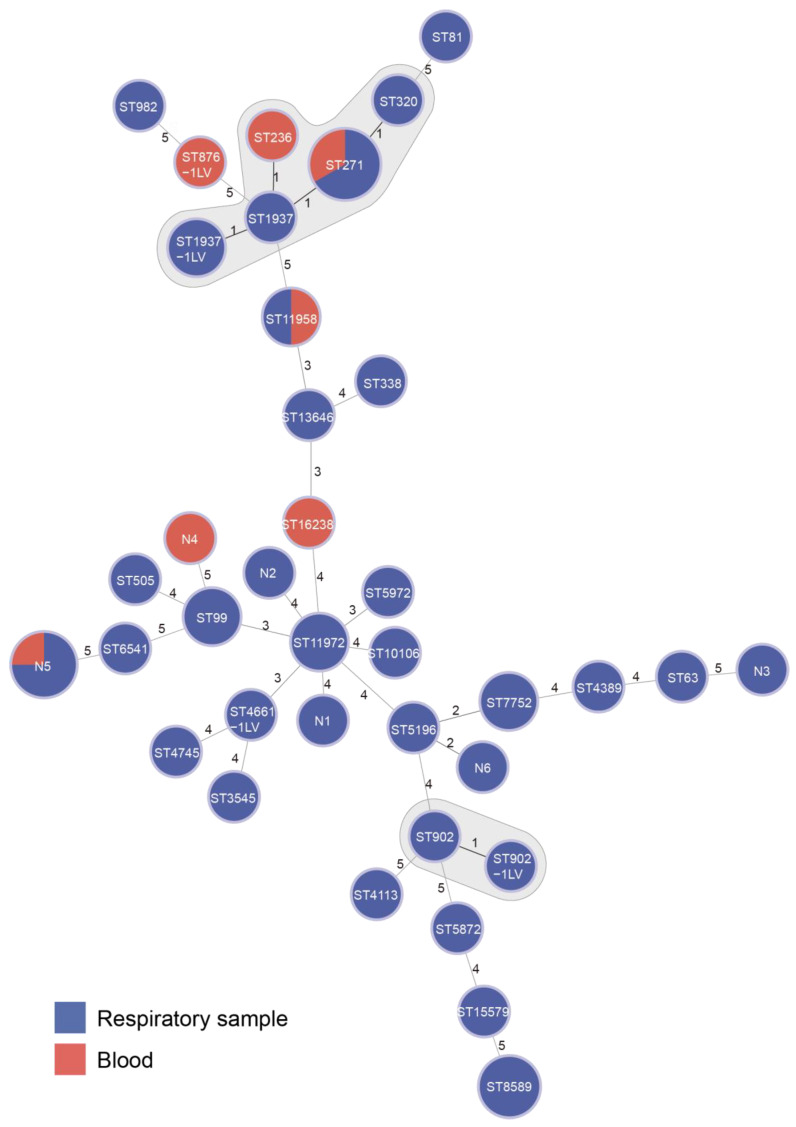
Relationships of STs of *S. pneumoniae* isolates in this study. Minimum spanning tree was constructed using Phyloviz software by the eBURST algorithm. The numbers on the circles represent the STs, the isolates cultured from respiratory samples are in blue and those from blood are in red. The clonal complexes are marked in shadow. The numbers on the lines represent the number of different loci between two STs.

**Figure 3:**
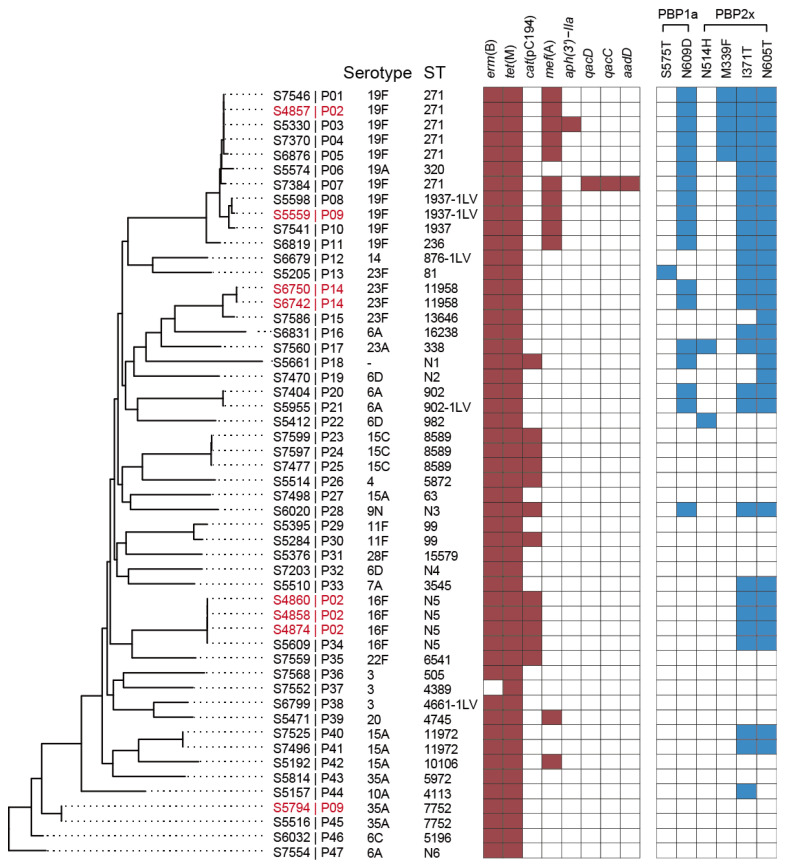
Phylogenetic analysis of *S. pneumoniae* isolates in our hospital correlated with the distribution of resistance genes (red–white heatmap) and resistance mutations (blue–white heatmap). The serotypes and STs of the isolates are also displayed. The patient numbers are attached after the isolate numbers, and the patients with more than one isolate are in red. N1 to N6 stand for the six new STs we identified.

By whole-genome sequencing, we obtained the genome sequences of the 52 *S. pneumoniae* isolates. The STs and serotypes were predicted based on the genome sequences. In total, 37 STs were identified (Figure 2), with 1–6 isolates per ST. Clonal complex (CC) 271 was the most common (21.2%, 11/52), consisting of ST271 (six isolates), ST320 (one isolate), ST1937 (one isolate), ST1937 single-locus variant (ST1937-1LV, two isolates) and ST236 (one isolate). Meanwhile, 21 serotypes were predicted and 1 isolate failed to be predicted (Figure 3), with 1–10 isolates per serotype and 19F (10 isolates) being the most common. Among these isolates, 49 were different strains from different patients. There were no SNPs between S6742 and S6750 from P02, and there were 1–4 SNPs among S4858, S4860 and S4874 from P14, with these isolates belonging to the same strains. Among these strains and serotypes from different patients, only 23 strains (46.9%, 23/49) of 7 serotypes of 3, 4, 6A, 14, 19A, 19F and 23F were covered by PCV13, and 23 strains of 9 serotypes of 3, 4, 9N, 10A, 14, 19A, 19F, 22F and 23F were covered by PPV23, which are the two vaccines currently used in China.

Phylogenetic analysis revealed the isolates were of large diversity, corresponding with the relationships of the STs that the isolates belonged to. Those of the same STs and CCs were clustering together, and the others of different STs were phylogenetically distinct from each other. However, the serotypes of the isolates were not always in accordance with the STs and phylogenetic relationships. The serogroup 19 (serotypes 19A and 19F) was only identified in CC271, and isolates belonging to CC271 and serogroup 19 were the most common, indicating that this group might be the most prevalent. In contrast, serogroup 6 (serotypes 6A, 6C and 6D), 15 (serotypes 15A and 15C) and 23 (serotypes 23A and 23F) were all identified in two or more phylogenetically distinct STs.

### 3.3. Carriage of Resistance Genes and Mutations

In line with the phenotypic characteristics of the antimicrobial susceptibility, multiple resistance genes were identified from the genome sequences. The most common were *tet*(M)-positive in all of the isolates conferring resistance to tetracyclines, and *erm*(B)-positive in 51 (98.1%) isolates conferring resistance to macrolides, lincosamides and streptogramin B (MLSB phenotype). Gene *cat* was positive in 12 (23.1%) isolates of several STs conferring resistance to chloramphenicols, and *mef*(A) was positive in 10 of the 11 CC271 isolates, with the other 2 isolates conferring resistance to macrolides. In addition, resistance-associated point mutations were identified in 32 isolates located in 2 penicillin-binding proteins, PBP1a and PBP2×, conferring non-susceptibility to penicillin. The most common amino acid mutations included N605T in PBP2× in 30 isolates (57.2%), I371T in PBP2× in 28 isolates (53.8%) and N609D in PBP1a in 18 isolates (34.6%). A total of 19 isolates carried mutations in both of the PBPs, including all of the 11 CC271 isolates. Among the 21 PRSP isolates, 19 (90.5%) carried mutations in PBPs, indicating that the mutations were significantly associated with the non-susceptibility to penicillin (*p* < 0.05).

### 3.4. Healthcare-Associated Infections and Cases Infected by Multiple Isolates

Although *S. pneumoniae* is the most common bacterial pathogen for CAIs, we found 15 infection cases occurring in the hospital, which were defined as HAIs (Table 1). However, by clinical investigation of the related cases in the same ward, we did not find any epidemiological relationships among the cases infected by isolates of the same STs. Therefore, we cannot confirm any nosocomial transmissions of the HAI cases.

In addition, from three patients we obtained more than one isolate. From patient P02, we obtained four isolates, including one ST271 serotype 19F isolate cultured from blood, and three serotype 16F isolates of a novel ST cultured from bronchial aspirate, blood in conduit or bronchoalveolar lavage fluid. The systematic infection occurred three months after admission, and the isolates were obtained within five days. From patient P14 (suffering from acute respiratory distress syndrome and septic shock), we also obtained two ST11958 serotype 23F isolates from peripheral blood and sputum within three days, respectively. From patient P09, we obtained a ST1937-1LV serotype 19F isolate and a ST7752 serotype 35A isolate, both from sputum samples, but the second isolation occurred two months later during a subsequent infection. These results indicated that infections might be associated with different isolates of different STs or serotypes at the same time, or successively.

## 4. Discussion

*S. pneumoniae* is the most common respiratory pathogen in children and older people. The diversity of this bacterium is extremely large, revealed by the large number of STs and serotypes [30]. Particularly, it can colonize on the mucosal surface of the upper airways amongst heathy people of all age groups, which are the sources of both CAIs and HAIs. In this study, we investigated the phenotypic resistance characteristics and molecular characteristics of *S. pneumoniae* in our hospital over a period of four years by whole-genome sequencing and analysis. Our results provided epidemiological information, which is meaningful for adjusting vaccine use and treatment strategies.

Although 37 STs were identified in our isolates, the CC271 was the dominant subtype, which is in line with previous studies [31,32]. The serotype prediction and analysis indicated that the PCV13 and PPV23, the two vaccines currently used in China, only covered a fraction of the serotypes we identified. Some less common serotypes are noteworthy, including serotype 6D, 15A, 15C and 16F, which are not covered by PCV13 and PPV23. Serotype replacement and the emergence of new serotypes could reduce the effectiveness of the vaccine. We also observed the high potential of serogroups 6, 15 and 23 to recombine amongst phylogenetically distinct STs, which indicated that we should pay more attention to the serotype variation of these serogroups.

Our results also demonstrated the serious antimicrobial resistances of *S. pneumoniae*, with high proportions of MDR and PRSP isolates. The carriages of *erm*(B), *tet*(M) and mutations in PBPs, including PBP1a and PBP2×, were remarkably high, corresponding to the high resistance rates to erythromycin (98%), tetracycline (96%) and penicillin G (42%). Fortunately, the isolates showed high susceptibility to fluoroquinolones. The resistance rates of levofloxacin (2%), ofloxacin (2%) and moxifloxacin (0%) were all quite low. These results indicated that fluoroquinolones would be effective. In contrast, the isolates showed certain resistance levels to cefotaxime (10%), amoxicillin (14%), ceftriaxone (14%) and chloramphenicol (18.8%), indicating the risks of treatment failure by these antimicrobials.

We also observed HAIs and cases infected by different subtypes of isolates. Since we did not find other cases infected by the same subtypes as those that infected the HAI cases, these cases might be infected by the isolates colonized in themselves or other patients not infected in the same wards, or even healthcare workers. Considering the high colonization rate of *S. pneumoniae* in healthy people, and the rapid transmission through the respiratory tract, the nosocomial transmissions and infections might be more common. 

In this study, there were several limitations. Since it was a retrospective study and the number of isolates was limited, we could not reveal more about the changes in the molecular subtypes and resistance characteristics of the isolates in different patient groups or over time. We therefore focused more on the molecular epidemiology of the isolates and less on the diseases of the patients, since the information was limited. In addition, as this was a single-center study, the results might be more representative of the local population, meaning more studies and results from other institutions are necessary to reveal an overall epidemiology of S. *pneumoniae* infection in the region.

## 5. Conclusions

In summary, we performed a retrospective analysis of the characteristics of the resistance phenotypes, genes and molecular subtypes of *S. pneumoniae* isolates in our hospital over a period of four years. The high carriages of resistance genes *erm*(B) and *tet*(M), and mutations in PBPs, highlight the importance of systematic surveillance. The clonal complex 271 serogroup 19 was the most prevalent subtype, exhibiting MDR phenotypes with a high carriage of resistance genes and mutations in PBPs. Our results called for further active surveillance of these subtypes, as well as the continuous optimization of the treatment protocols based on the surveillance.

## Figures and Tables

**Table 1:** Information relating to the *S. pneumoniae* isolates and patients.

Isolate	Sample	Isolation Year	Patient	Age of Patient	Sex of Patient	Infection Type *	Underlying Diseases
S7546	throat swab	2022	P01	6	M	CAI	sleep apnea
S4857	peripheral blood	2018	P02	68	M	HAI	abdominal cocoon
S5330	peripheral blood	2018	P03	74	M	HAI	brain tumor
S7370	throat swab	2021	P04	7	M	CAI	adenoid hypertrophy
S6876	sputum	2020	P05	69	M	HAI	pancreatic tumor
S5574	sputum	2019	P06	88	M	HAI	lung cancer
S7384	throat swab	2021	P07	4	M	CAI	sleep apnea
S5598	nasopharyngeal swab	2019	P08	46	M	CAI	sleep apnea
S5559	sputum	2019	P09	61	M	HAI	nephrotic syndrome
S7541	sputum	2022	P10	5	F	CAI	bronchopneumonia
S6819	peripheral blood	2020	P11	78	M	CAI	lung infection
S6679	peripheral blood	2020	P12	48	F	HAI	liver cirrhosis
S5205	sputum	2018	P13	72	M	CAI	cerebral infarction
S6750	peripheral blood	2020	P14	30	F	CAI	septic shock
S6742	sputum	2020	P14	30	F	CAI	septic shock
S7586	throat swab	2022	P15	5	M	CAI	sleep apnea
S6831	peripheral blood	2020	P16	50	M	HAI	acute cholangitis
S7560	throat swab	2022	P17	5	M	CAI	sleep apnea
S5661	sputum	2019	P18	71	M	CAI	lung infection
S7470	throat swab	2022	P19	4	M	CAI	adenoid hypertrophy
S7404	throat swab	2022	P20	5	M	CAI	secretory otitis media
S5955	sputum	2019	P21	60	M	HAI	cerebral infarction
S5412	sputum	2018	P22	64	M	HAI	cerebral infarction
S7599	throat swab	2022	P23	5	M	CAI	adenoid hypertrophy
S7597	throat swab	2022	P24	4	F	CAI	sleep apnea
S7477	sanies	2022	P25	5	M	CAI	adenoid hypertrophy
S5514	sputum	2019	P26	85	M	CAI	lung infection
S7498	sputum	2022	P27	-	-	-	unknown
S6020	sputum	2019	P28	67	M	CAI	severe pneumonia
S5395	sputum	2018	P29	84	F	HAI	cerebral infarction
S5284	sputum	2018	P30	78	M	CAI	cerebral infarction
S5376	sputum	2018	P31	69	M	CAI	lung infection
S7203	peripheral blood	2021	P32	70	M	CAI	suppurative meningitis
S5510	bronchial aspirate	2019	P33	85	M	CAI	lung infection, urinary tract infection
S4860	bronchial aspirate	2018	P02	68	M	HAI	abdominal cocoon
S4858	blood in conduit	2018	P02	68	M	HAI	abdominal cocoon
S4874	bronchoalveolar lavage fluid	2018	P02	68	M	HAI	abdominal cocoon
S5609	sputum	2019	P34	71	M	CAI	esophageal cancer
S7559	bronchoalveolar lavage fluid	2022	P35	69	M	CAI	lung cancer
S7568	sputum	2022	P36	30	M	CAI	lung infection
S7552	sputum	2022	P37	68	M	CAI	type II respiratory failure
S6799	pleural effusion	2020	P38	74	F	CAI	esophageal cancer
S5471	sputum	2018	P39	75	F	CAI	rib fracture
S7525	sputum	2022	P40	48	M	CAI	acute-on-chronic liver failure
S7496	bronchoalveolar lavage fluid	2022	P41	82	M	CAI	lung infection
S5192	sputum	2018	P42	72	M	CAI	cerebral infarction
S5814	sputum	2019	P43	66	M	CAI	cerebral infarction
S5157	sputum	2018	P44	-	-	-	
S5794	sputum	2019	P09	61	M	HAI	nephrotic syndrome
S5516	sputum	2019	P45	97	M	HAI	cerebral hemorrhage
S6032	sputum	2019	P46	74	M	CAI	cerebral infarction
S7554	throat swab	2022	P47	4	F	CAI	adenoid hypertrophy

* CAI: community-acquired infection; HAI: healthcare-associated infection.

## Data Availability

All sequencing data have been deposited in the NCBI database under accession number PRJNA898686 (https://www.ncbi.nlm.nih.gov/bioproject/PRJNA898686, accessed on 8 January 2023).
